# Safety of FEES Performed by Speech-Language Pathologists and Physicians–Evidence Supporting Task Sharing from a Retrospective Observational Study of 964 Consecutive Examinations

**DOI:** 10.3390/nu17203193

**Published:** 2025-10-10

**Authors:** Małgorzata Polit, Joanna Chmielewska-Walczak, Maria Sobol, Izabela Domitrz, Kazimierz Niemczyk

**Affiliations:** 1 Department of Neurology, Faculty of Medicine and Dentistry, Medical University of Warsaw, 02-091 Warsaw, Poland; malgorzata.polit@wum.edu.pl (M.P.);; 2 Department of Otorhinolaryngology, Head and Neck Surgery, Faculty of Medicine, Medical University of Warsaw, 02-091 Warsaw, Poland; 3 Department of Biophysics, Physiology and Pathophysiology, Medical University of Warsaw, 02-004 Warsaw, Poland

**Keywords:** deglutition disorders, patient safety, risk assessment, speech-language pathology

## Abstract

(1) **Background:** Fiberoptic Endoscopic Evaluation of Swallowing (FEES) is one of the two gold-standard tools for assessing oropharyngeal dysphagia (alongside Videofluoroscopic Swallowing Study). Although generally considered safe, concerns about complications persist, particularly in systems where FEES is not routine and professional roles differ. The aim of this study was to evaluate the safety of FEES performed by both speech-language pathologists (SLPs) and physicians, in order to provide evidence of its safety in a healthcare system where the procedure is not yet widely established and to identify patient subgroups potentially at higher risk of procedure-related complications. (2) **Methods:** This retrospective study analyzed 964 consecutive FEES procedures. Examinations were carried out by trained SLPs or physicians. Data included demographics, clinical status, operator qualifications, setting, and complications, classified as minor (vomiting, poor tolerance, early termination) or major (laryngospasm, epistaxis). (3) **Results:** The overall complication rate was 1.14% (11/964): 0.6% minor and 0.5% major. All events were self-limiting. Complication rates did not differ between SLPs (1.05%) and physicians (1.23%) or by experience, setting, drug use, penetration–aspiration scale score, or nasogastric tube. Four complications occurred in amyotrophic lateral sclerosis patients, suggesting higher risk. (4) **Conclusions:** FEES is safe and well tolerated when performed by either physicians or SLPs. These findings underscore the value of task sharing in dysphagia diagnostics, demonstrating that a shared model increases service capacity, reduces delays, and facilitates timely management of dysphagia.

## 1. Introduction

Diagnosis of oropharyngeal dysphagia follows a stepwise approach. The initial assessment typically involves screening tests, disease-specific diagnostic questionnaires, patient-reported quality-of-life measures, and a focused clinical swallowing evaluation. These approaches are relatively simple to apply and do not require advanced equipment, which makes them widely accessible in clinical practice. Although widely applied, the supporting evidence for patient benefit is still limited. For this reason, instrumental assessment including Fiberoptic Endoscopic Evaluation of Swallowing (FEES) and Videofluoroscopic Swallowing Study (VFSS) is considered the diagnostic gold standard, as it enables direct visualization of penetration and aspiration and delineation of the underlying swallowing pathophysiology [1,2].

FEES, introduced by speech-language pathologist (SLP) S.E. Langmore in the late 1980s [3,4,5,6], offers several advantages: it can be performed at the bedside, even for patients with limited mobility or reduced cooperation; it can be repeated or extended as needed; and it enables the assessment of secretion management and laryngeal sensation—all without radiation exposure. FEES plays a key role in the adaptive and compensatory therapy of dysphagia, as it provides immediate recommendations on dietary texture, bolus size, and fluid consistency, forming the basis for safe feeding strategies [7]. It also supports biofeedback-based training in compensatory swallowing strategies [4,8].

Although FEES is generally considered a safe and well-tolerated procedure, concerns remain about potential complications such as gagging, vomiting, epistaxis, laryngospasm, or vasovagal syncope [8,9,10,11,12,13,14,15]. While in some countries it is common practice for SLPs to independently perform FEES, this is not universally accepted across many other regions. One of the main arguments is the concern regarding the safety of the procedure when performed by non-physicians. Previous studies examining the safety of FEES have called for further evaluation in health systems where the procedure is still being introduced, particularly when performed by less experienced clinicians [15]. This study directly addresses this gap by providing real-world safety data from a setting in which FEES is not yet performed routinely by either SLPs or physicians and is currently limited to a small but growing number of centers.

This lack of access and regulations contributes to delays in the diagnosis, appropriate management and decision-making of oropharyngeal dysphagia, a condition associated with malnutrition, dehydration, aspiration pneumonia, difficulty with medication intake, reduced quality of life, and increased mortality—posing significant risks to affected patients [16,17,18,19,20]. Dysphagia is also associated with increased healthcare costs, due to the management of its complications [21,22,23]. Incorporating FEES into clinical care has been shown to improve dysphagia management and outcomes. It is also crucial for optimizing nutrition in patients [14].

The aim of this study was to provide evidence on the safety of FEES performed by both physicians and SLPs, thereby supporting the development of collaborative care models. Task sharing may help expand access to FEES in health systems where the procedure is not yet routinely performed and clinical experience remains limited.

A secondary objective was to identify patient subgroups potentially at higher risk of procedure-related complications.

## 2. Materials and Methods

### 2.1. Study Design and Setting

A retrospective observational study was conducted on 964 consecutive FEES examinations performed in 760 patients between January 2019 and September 2024 as part of routine clinical care. Data were collected from two clinics at the Medical University of Warsaw: the Department of Neurology and the Department of Otorhinolaryngology, Head and Neck Surgery, each located in a separate multidisciplinary hospital.

### 2.2. Examiner Qualifications and Procedural Roles

Examinations were performed by one of two SLPs who had completed certified FEES training provided by the European Society for Swallowing Disorders (ESSD), or by physicians exclusively from the specialties of neurology, otolaryngology, and phoniatrics [24]. Among the physicians, only one had completed the ESSD instructor-level course. Although the SLPs and neurologists had no prior experience inserting a fiberoptic endoscope, the otolaryngologists and phoniatricians had each conducted more than 100 endoscopic examinations before the study period. Procedures were conducted either independently or with the assistance of a physician or another SLP, depending on staffing and clinical circumstances.

### 2.3. Patient Characteristics

Both hospitalized and outpatient populations were included in the study. FEES was performed in patients referred by a physician following clinical evaluation. Referrals originated primarily from neurologists, otolaryngologists, and phoniatricians, as well as from physicians in departments of internal medicine, gastroenterology, cardiology, neurosurgery, and the intensive care unit (ICU).

Some referrals involved patients undergoing pre- or postoperative assessment related to head and neck cancer procedures, in whom dysphagia needed to be clinically ruled out. In certain cases, FEES was necessary to definitively exclude oropharyngeal dysphagia during the diagnostic process. As a result, the dataset included examinations of patients without confirmed oropharyngeal dysphagia. However, the absence of dysphagia was not considered an exclusion criterion, as the study aimed to include all consecutive FEES examinations performed during the study period, regardless of the final diagnosis.

The only exclusion criterion based on patient characteristics was age under 16 years, because individuals below this age are not legally permitted to provide informed consent for medical procedures under Polish law.

### 2.4. Analyzed Data

The retrospective analysis included data extracted from medical records and FEES documentation. Patient-related variables encompassed age, sex, and primary diagnosis. The presence or absence of oropharyngeal dysphagia was recorded, along with indicators of clinical complexity such as respiratory dysfunction and reduced level of consciousness. Additional data included care setting (inpatient or outpatient), acuity of the patient’s condition at the time of examination (acute or stable), and the specific location where FEES was performed. Clinical risk factors potentially affecting procedural safety were also collected, including history of pneumonia, laryngeal or vocal fold paresis, sensory deficits in the laryngeal area, and the use of enteral nutrition. The presence of a nasogastric tube and any associated mucosal damage were documented, along with unintentional weight loss and the presence of voice or speech disorders. The use of anticoagulant or antiplatelet medications at the time of the procedure was also noted.

Examiner-related data included the professional role of the individual performing the examination—either an SLP or a physician—as well as their level of experience, categorized as fewer than 30 or 30 or more FEES procedures. It was also noted whether the examination was performed independently or with the assistance of another clinician, such as a physician or another SLP.

Procedure-specific variables included the use of sensory testing during the examination, defined as direct endoscopic stimulation of laryngeal structures using the tip of the endoscope. Additionally, penetration-aspiration scale (PAS) scores were recorded, and the presence of saliva penetration or aspiration at the start of the examination was noted [25].

All complications were reviewed and classified by severity. Minor complications included gagging, vomiting, minor mucosal injury, and early termination of the examination due to poor tolerance. Major complications were defined as epistaxis, laryngospasm, vasovagal syncope, or airway compromise [15].

### 2.5. FEES Protocol

A flexible video nasopharyngoscope (XION Endo Flex; 3.5 mm diameter, XION GmbH, Berlin, Germany) equipped with an integrated camera, light source, optics, and microphone was used for the majority of examinations. The endoscope tip was lubricated with oil prior to nasal insertion; no topical anesthesia was used.

All examinations strictly followed the standard FEES protocol developed by S.E. Langmore [8]. In addition, all examiners completed the same FEES training accredited by the ESSD, which ensured comparable preparation for performing the protocol and conducting the procedure.

Patients received a detailed explanation of the procedure. Medical history and reported symptoms were reviewed, followed by a brief anatomical and functional assessment of the oropharyngeal structures, including cranial nerve screening. Depending on clinical condition, patients were seated upright in a chair or bed. Food and water were dyed for visibility. Liquids were thickened with a gum-based thickener, and consistencies were prepared according to the International Dysphagia Diet Standardisation Initiative framework [26], typically stages 0, 2, and 4 (liquids) and stages 5 and 7 (solids). Swallowing safety was assessed using the PAS scale [25].

The protocol included three phases. In the initial phase, an anatomical and functional evaluation was performed after endoscope insertion, assessing velopharyngeal closure, spontaneous swallowing, saliva management, phonation, cough strength, breath hold, tongue base retraction, laryngeal elevation, pharyngeal constriction, and laryngeal sensation.

The second phase involved swallowing trials with boluses of varying volumes and consistencies, assessing oral control, initiation of swallowing, epiglottic retroflexion, penetration/aspiration events, and bolus clearance.

The final phase involved compensatory strategies aimed at improving both the safety and efficacy of swallowing. Patients were often trained in these techniques with the aid of real-time biofeedback. This comprehensive approach enabled the identification of dysphagia mechanisms, severity grading, and individualized nutritional recommendations.

### 2.6. Statistical Analysis

Data were analyzed using Statistica 13.3 (data analysis software system), version 13.6.0 (TIBCO Software Inc., Santa Clara, CA, USA). Categorical variables were expressed as percentages, while continuous variables were presented using descriptive statistics (mean ± standard deviation, median, and range). The Chi-square test or Fisher’s exact test was used to compare categorical variables. Results were considered statistically significant if the *p*-value was less than α = 0.05.

## 3. Results

The study analyzed a total of 964 consecutive FEES procedures. All percentages and frequencies presented in this section refer to the number of examinations rather than individual patients, because several patients underwent multiple assessments during the study period.

In the study group, 50.7% of procedures (*n* = 481) were performed on male patients and 49.3% (*n* = 475) on female patients. The mean patient age at the time of examination was 61 ± 17 years, with a median of 65 and a range of 16–97 years.

Pharyngeal dysphagia was identified in 81.6% of examinations, while esophageal dysphagia was identified in 1%, and no dysphagia was observed in 17.4%.

FEES was most commonly performed in patients with head and neck cancer (36.7% of examinations), followed by other conditions (23.3%). Within the neurological group, stroke was the most frequently observed diagnosis (14.6%). The next subgroup comprised chronic neurological disorders (12.3%), including myasthenia gravis, ALS, Parkinson’s disease, progressive supranuclear palsy, Bickerstaff’s brainstem encephalitis, spinocerebellar ataxia, dystonia, and post–status epilepticus (Table 1).

Moreover, in the largest group of cases, 3.1% (30 examinations) involved pre-existing mucosal injury of the hypopharynx unrelated to the procedure, while 2.2% (21 examinations) showed mucosal injury in the mid-pharynx. Aphasia was noted in 42.4% of examinations (*n* = 409), voice disorders in 29.9% (*n* = 288), and dysarthria in 18.7% (*n* = 180).

A total of 55.4% of examinations (*n* = 534) were conducted during hospital stays, while 44.6% (*n* = 430) were performed in outpatient settings. Among the in-hospital procedures, 47.6% (*n* = 254) were performed in patients admitted through regular admission and 53.4% (*n* = 285) in patients receiving acute care.

The FEES procedure was most frequently performed in the otolaryngology department, accounting for 80.9% of examinations (*n* = 780), followed by the stroke department (15.6%, *n* = 150), the neurology department (3.4%, *n* = 33), and the ICU (0.1%, *n* = 1). In 80.9% of cases (*n* = 809), the examination was performed in an outpatient office setting, whereas 16.1% were conducted at the bedside. In 40.5% of examinations (*n* = 391), the procedure was performed by a physician, in 29.5% (*n* = 285) by an SLP, and in 30% (*n* = 288) by a team consisting of both. Among these team procedures, the endoscope was operated by the SLP in 66% (190) and by the physician in 34% (98). Patient cooperation during the procedure was classified as good in 87.9% of examinations (*n* = 847) and as impaired in 12.1% (*n* = 117). Limited cooperation was mainly due to neurological deficits, particularly cognitive impairments, which reduced patients’ ability to respond promptly and accurately and to follow instructions during the examination.

Complications were reported in 11 cases (1.1%), including laryngospasm (*n* = 4), vomiting (*n* = 3), and epistaxis (*n* = 1). No mucosal damage, respiratory complications, vasovagal episodes, or adverse reactions to food dye were observed. Three procedures had to be discontinued before completion. No adverse effects requiring additional medical intervention occurred following any FEES examination. All reported complications were self-limiting and resolved spontaneously within a short period.

The laryngeal touch sensation test was successfully performed in 887 of 925 applicable examinations (95.9%). A nasogastric tube was present during 128 examinations.

At the time of the FEES examination, 40 patients (4.1%) were experiencing active pneumonia. These were cases of hospital-acquired pneumonia, including aspiration pneumonia.

A total of 39 examinations (4.0%) were performed in patients with a history of total laryngectomy. Unilateral laryngeal paralysis was documented in 220 examinations (22.8%), bilateral paralysis in 52 (5.4%), and no paralysis in 653 (67.7%). Impaired laryngeal sensation was observed in 484 examinations (52.3%), while 441 (47.7%) showed no sensory deficit.

Most procedures (*n* = 730; 75.7%) were performed in patients receiving oral nutrition, followed by 148 with nasogastric tube feeding (15.4%), 79 with percutaneous endoscopic gastrostomy (8.2%), 4 with parenteral nutrition (0.4%), and 3 with mixed feeding (0.3%). Unintentional weight loss was recorded in 125 examinations (13.0%). Salivary penetration or aspiration at baseline was observed in 214 procedures (22.2%).

To evaluate the correlation between FEES complications and various factors—including the operator’s level of experience, the individual performing the procedure, the procedural setting (bedside vs. outpatient clinic), the examination location (hospital ward), the procedural environment (hospital vs. outpatient facility), the use of antiplatelet or anticoagulant medications, and the presence of a nasogastric tube—either the Chi-square test or Fisher’s exact test was used, as appropriate. No statistically significant differences were found in any of the comparisons.

Regarding the location of the examination, the highest incidence of side effects was observed in the neurology unit, where 6.1% (2 patients) experienced complications. However, after adjustment for multiple comparisons, this result did not reach statistical significance (summary in Table 2).

For the primary diagnosis, the *p*-value approached the threshold of statistical significance. Among diagnostic categories, patients with chronic neurological disorder had the highest number of side effects, with 3.9% (5 patients) affected. Four complications in this group occurred in patients with ALS. In the group of patients with ALS, the observed complications included one case of laryngospasm, two episodes of vomiting, and one early termination of the procedure due to poor tolerance and gagging. All patients in this subgroup presented with bulbar and pseudobulbar signs. Two examinations were performed in patients with isolated bulbar muscle involvement, while the remaining patients also exhibited limb-related neurological deficits.

Additionally, the relationship between dysphagia severity, measured by the worst penetration–aspiration scale (PAS) score during FEES, and the occurrence of complications was evaluated. No significant differences were observed (*p* = 0.302). Complications were rare across all PAS categories, ranging from 0% at scores 0, 3, and 5, to between 0.56% and 2.22% at scores 1, 2, 4, and 6. The highest rates were noted at PAS 7 (4.11%) and PAS 8 (1.67%). Overall, the incidence of complications remained low and showed no statistically significant association with dysphagia severity as measured by the PAS scale.

## 4. Discussion

In line with previous reports, the present study confirms that FEES is associated with a low complication rate. The overall complication rate observed was 1.14%. Among these complications, six were classified as minor (three cases of vomiting and three premature terminations due to poor tolerance), and five as major (one case of self-limiting epistaxis and four instances of laryngospasm). No cases of allergic reaction to food dye, vasovagal syncope, or airway compromise were recorded. The low complication rate may, in part, reflect the standardized training provided to examiners by the ESSD. Furthermore, the consistency of these findings with previously reported complication rates—typically ranging from 1% to 2%—supports their external validity [8,9,10,11,12,13,14,15].

No statistically significant differences were observed based on the examiner’s professional background or level of endoscopic experience. These findings support the conclusion that appropriately trained SLPs can safely perform FEES, with a complication profile comparable to that reported internationally. This supports the concept of task sharing, in which clinical responsibilities are distributed across professional groups to expand service capacity. Such models are particularly relevant in healthcare systems with limited access to instrumental dysphagia diagnostics, as they can improve patient access to timely assessment and management. This is particularly important in relation to the studied patient groups, who require a diagnostic work-up that goes beyond standard tools such as screening tests, questionnaires, or clinical swallowing evaluations. For example, in patients in the acute phase of stroke, the detection rate of dysphagia using screening is 37–45%, with clinical swallowing evaluations 51–55%, whereas with instrumental assessment the rate increases to 64–78% [27]. This clearly demonstrates the necessity of access to instrumental diagnostics, such as FEES, in the care of stroke patients. Such recommendations are also reflected in the guidelines developed by the European Stroke Organisation and the ESSD regarding post-stroke dysphagia management [28]. Similarly, in patients with head and neck cancer, anatomical and sensory changes resulting from surgery or radiotherapy require direct evaluation of swallowing function through instrumental assessments. As highlighted in the White Paper of the ESSD, clinical scales alone are often insufficient or not validated for this population, highlighting the need for access to instrumental diagnostics [29]. Broadening access to FEES diagnostics, performed by different clinician groups, can therefore help ensure better care for these patient groups. Our results align with the findings of Dziewas et al. [14], who likewise demonstrated that FEES can be safely performed across different clinician groups, including those with relatively limited endoscopic experience.

Most prospective studies on FEES safety focus on patient-reported discomfort during the procedure. However, this parameter is not routinely included in the standard FEES protocol and was, therefore, not assessed in the present retrospective analysis. Previous reports indicate that over 81% of patients rate the procedure as only mildly to moderately uncomfortable, with severe discomfort being rare, suggesting a generally high level of tolerance [9,10,12]. In the current study, only three procedures were prematurely terminated due to patient discomfort, including gag reflex and low procedural tolerance. Although the intensity of discomfort could not be quantified, the low discontinuation rate supports the conclusion that FEES was generally well tolerated by patients.

Vomiting during FEES has been reported in approximately 2% of patients in previous studies [13]. In the present study, it occurred in only 0.3% of cases. Although classified as a minor complication, vomiting can significantly affect patient comfort, contribute to reluctance toward future examinations, and may necessitate early termination before the full protocol is completed. Potential contributing factors include an exaggerated gag reflex or patient anxiety. Therefore, it is essential to provide a clear explanation of the procedure beforehand and maintain continuous communication with the patient during the examination. This allows for behavioral guidance, such as minimizing head movements, which can help prevent endoscope contact with the pharyngeal wall and reduce the risk of triggering gagging or vomiting reflexes.

Reports of epistaxis during FEES vary, with some studies reporting rates as high as 6% [12]. In the present study, only one case (0.1%) of nasal bleeding occurred, which resolved spontaneously without medical intervention. While a previous study mentioned using a water-based lubricant, this study employed an oil-based lubricant to ease endoscope insertion through the nasal cavity. This approach may be especially beneficial, as many patients present with dry or damaged nasal mucosa due to dehydration, oxygen therapy, or prior nasogastric tube use. Applying a suitable lubricant to the distal end of the endoscope may help reduce friction and minimize mucosal injury.

Laryngospasm occurred in four cases (0.4%) in this study. One episode took place during a procedure performed by an SLP in a patient with ALS, while the remaining three occurred during physician-led examinations: one in a patient with head and neck cancer, one in a post-stroke patient with chronic oropharyngeal dysphagia, and one in a patient with a neurodegenerative disorder. These episodes happened during penetration or aspiration of liquids or solids or during sensory testing—specifically when the physician stimulated the subglottic area with the endoscope tip. Previous studies have also reported a low incidence of laryngospasm (0.3–0.8%), with events being self-limiting and not requiring further intervention [8,14,15]. As emphasized by S.E. Langmore, direct contact between the endoscope and the larynx—particularly the vocal folds—should be avoided. She notes that stimulation of the aryepiglottic folds and epiglottis poses minimal risk of triggering laryngospasm [8]. Strict adherence to the FEES protocol is crucial to ensuring patient safety.

No significant association was found between dysphagia severity, as measured by the PAS, and the occurrence of complications during FEES. Even among patients with high PAS scores (7–8), complications were infrequent. This suggests that dysphagia severity, as measured by PAS, may not be a strong predictor of adverse events during the FEES procedure.

This study presents the first and largest retrospective analysis to date of FEES procedures performed by SLPs and physicians in the country where the method is not yet widely implemented and clinical centers are only beginning to gain experience, which constitutes the main strength of this study. By including both inpatient and outpatient populations with a broad range of medical conditions—particularly neurological disorders and head and neck cancer—this study enabled a comprehensive safety assessment of FEES in clinically vulnerable groups at elevated risk of complications. A major strength lies in the use of a consecutive, non-selective recruitment strategy, which minimized selection bias and enhanced the generalizability of the findings to routine clinical practice.

Several limitations should be acknowledged. First, substantial missing data for certain variables, due to inconsistent documentation by examiners, may have affected the internal validity of the findings. Second, the retrospective design and reliance on routine medical records may have led to underreporting of minor complications, potentially underestimating the true incidence. Additionally, examiner-related factors—such as procedural training, interdisciplinary collaboration, and institutional protocols—were not standardized and may have influenced complication rates. Prospective, multicenter studies with standardized documentation protocols are needed to confirm these findings and strengthen the evidence base.

Some studies on the safety of FEES have noted that patients with ALS tend to tolerate the procedure less well and may be at higher risk for major complications such as laryngospasm [9,13,15]. In our study, 4 of the 11 recorded complications occurred in patients diagnosed with ALS (Table 1.), all of whom presented with bulbar and pseudobulbar signs. Information regarding this specific patient subgroup has been limited in previous studies and was typically restricted to the number of reported complications. These findings suggest that a more detailed and focused safety analysis of FEES is warranted in this patient population, highlighting the need for further dedicated research. Moreover, other reports, including studies based on a multidisciplinary diagnostic model in ALS, have shown that patients with bulbar-onset ALS develop speech and swallowing impairments earlier and more severely than those with the spinal form, due to the high vulnerability of tongue and pharyngeal musculature to neurodegeneration [30]. These findings support the hypothesis that ALS patients represent a particularly vulnerable subgroup for FEES, underscoring the importance of tailored FEES protocols. Prospective, multicenter studies with standardized documentation are needed to validate these observations and strengthen the evidence base.

## 5. Conclusions

This study confirms that FEES is a safe procedure when performed by appropriately trained SLPs or physicians, across both inpatient and outpatient populations with varying levels of medical complexity—even in healthcare systems where the procedure is not yet widely established or formally regulated. These findings support the continued implementation and broader adoption of FEES as a reliable tool to improve the management of patients with dysphagia. Our findings support collaborative task sharing in dysphagia diagnostics, showing that both physicians and SLPs can safely provide FEES. Such models of shared responsibilities may increase access to instrumental swallowing assessment, reduce diagnostic delays, and facilitate earlier decision-making.

Further research is warranted to examine the safety of FEES more closely in patients with ALS, who may represent a higher-risk population.

## Figures and Tables

**Table 1:** Complications during FEES according to primary diagnosis.

Primary Diagnosis	*n* (Exams)	*n* (%) of Total	Complications *n* (%)	Type and Number of Complications	Description of Patients Desease
Head and neck cancer	355	36.7%	3 (0.8%)	1 Laryngospasm, 1 Epistaxs, 1 Early termination of the procedure	3 patients after paraganglioma surgery
Other conditions	227	23.3%	1 (0.4%)	1 Vomiting	Postoperative state after skull base abscess
Stroke	171	14.6%	1 (0.6%)	1 Laryngospasm	A patient after two ischemic strokes in the chronic phase
Chronic neurological diseases	127	12.3%	5 (3.9%)	2 Laryngospasm, 2 Vomiting, 1 Early termination of the procedure	4 patients with ALS; 1 patient under diagnostic evaluation for a neurodegenerative disease
Pneumonia	11	1.1%	0 (0%)	–	–
Gastroesophageal reflux disease	17	1.8%	0 (0%)	–	–
Cardiac conditions	15	1.6%	1 (6.7%)	1 Early termination of the procedure	A patient after cardiac surgery with a history of thyroid cancer treated with radiotherapy
No diagnosis	31	3.2%	0 (0%)	–	–
Brain tumor	10	1.0%	0 (0%)	–	–
Total	964	100%	11 (1.1%)	4 laryngospasms, 3 vomiting, 1 epistaxis, 3 early termination of the procedure	–

**Table 2:** Factors associated with complications during FEES examinations.

Category	Analyzed Variables	No Complications (*n*, %)	Complications (*n*, %)	*p*-Value
Operator experience	Up to 30 procedures	70 (98.6%)	1 (1.4%)	0.823
	More than 30 procedures	883 (98.9%)	10 (1.1%)	
Procedure performer	Physician	482 (98.8%)	6 (1.2%)	0.796
	Speech-language pathologist	470 (99.0%)	5 (1.0%)	
Examination setting	Bedside	155 (100%)	0 (0%)	0.229
	Outpatient office	798 (98.6%)	11 (1.4%)	
Hospital unit	Stroke unit	150 (100%)	0 (0%)	0.024
	Neurology unit	31 (93.9%)	2 (6.1%)	
	Intensive care unit	1 (100%)	0 (0%)	
	Otolaryngology unit	771 (98.8%)	9 (1.2%)	
Nasogastric tube	No	825 (98.7%)	11 (1.3%)	0.192
	Yes	128 (100%)	0 (0%)	
Antiplatelet use	No data	756 (98.8%)	9 (1.2%)	0.355
	Yes	106 (100%)	0 (0%)	
	No	91 (97.9%)	2 (2.1%)	
Anticoagulant use	No data	755 (98.8%)	9 (1.2%)	0.431
	Yes	96 (100%)	0 (0%)	
	No	102 (98.1%)	2 (1.9%)	
Treatment setting	Hospital	528 (98.9%)	6 (1.2%)	0.955
	Outpatient	425 (98.8%)	5 (1.2%)	

## Data Availability

The raw data supporting the conclusions of this article will be made available by the authors on request.
